# Long-term Outcomes Associated With Permanent Pacemaker Implantation After Surgical Aortic Valve Replacement

**DOI:** 10.1001/jamanetworkopen.2021.16564

**Published:** 2021-07-13

**Authors:** Natalie Glaser, Michael Persson, Magnus Dalén, Ulrik Sartipy

**Affiliations:** 1 Department of Cardiology, Stockholm South General Hospital, Stockholm, Sweden; 2 Department of Molecular Medicine and Surgery, Karolinska Institutet, Stockholm, Sweden; 3 Department of Cardiothoracic Surgery, Karolinska University Hospital, Stockholm, Sweden

## Abstract

**Importance:**

Prior studies investigating the long-term clinical outcomes of patients who have undergone permanent pacemaker implantation after aortic valve replacement reported conflicting results.

**Objective:**

To investigate long-term outcomes after primary surgical aortic valve replacement among patients who underwent postoperative permanent pacemaker implantation.

**Design, Setting, and Participants:**

This cohort study included all patients who underwent surgical aortic valve replacement in Sweden from 1997 to 2018. All patients who underwent primary surgical aortic valve replacement in Sweden and survived the first 30 days after surgical treatment were included. Patients who underwent preoperative permanent pacemaker implantation, concomitant surgical treatment for another valve, or emergency surgical treatment were excluded. Patients who underwent concomitant coronary artery bypass grafting or surgical treatment of the ascending aorta were included. Follow-up data were complete for all patients. Data were analyzed from October through December 2020.

**Exposures:**

Patients underwent implantation of a permanent pacemaker or implantable cardioverter defibrillator within 30 days after aortic valve replacement.

**Main Outcomes and Measures:**

The primary outcome was all-cause mortality.

**Results:**

Among 24 983 patients who underwent surgical aortic valve replacement, 849 patients (3.4%) underwent permanent pacemaker implantation within 30 days after surgical treatment and 24 134 patients (96.6%) did not receive pacemakers in that time. The mean (SD) age of the total study population was 69.7 (10.8) years, and 9209 patients were women (36.9%). The mean (SD) and maximum follow-up periods were 7.3 (5.0) years and 22.0 years, respectively. At 10 years and 20 years after surgical treatment, the Kaplan-Meier estimated survival rates were 52.8% and 18.0% in the pacemaker group, respectively, and 57.5% and 19.6% in the nonpacemaker group, respectively. All-cause mortality was statistically significantly increased in the pacemaker group compared with the nonpacemaker group (hazard ratio [HR], 1.14; 95% CI, 1.01-1.29; *P* = .03), and so was risk of heart failure hospitalization (HR, 1.58; 95% CI, 1.31-1.89; *P* < .001). No statistically significant increase was found in the risk of endocarditis in the pacemaker group.

**Conclusions and Relevance:**

This study found that there were increased risks of all-cause mortality and heart failure hospitalization among patients who underwent permanent pacemaker implantation after surgical aortic valve replacement, suggesting that these risks are important considerations, especially in an era when transcatheter aortic valve replacement is used in younger patients at lower risk of adverse surgical outcomes. These findings further suggest that future research should investigate how to avoid permanent pacemaker dependency after surgical and transcatheter aortic valve replacement.

## Key Points

**Question:**

Is permanent pacemaker implantation after aortic valve replacement associated with long-term adverse clinical outcomes?

**Findings:**

In this cohort study of 24 983 patients who underwent surgical aortic valve replacement, increased risks of death and heart failure hospitalization were observed among patients who underwent permanent pacemaker implantation after aortic valve replacement compared with those who did not.

**Meaning:**

The association of mortality with permanent pacemaker implantation after aortic valve replacement should be considered, especially in an era when transcatheter aortic valve replacement is used among patients who are younger and have lower risks of adverse surgical outcomes.

## Teaser

This cohort study investigates the association of long-term adverse clinical outcomes, including mortality, with permanent pacemaker implantation after surgical aortic valve replacement.

## Introduction

Aortic valve replacement (AVR) is associated with radically improved prognosis among patients with severe aortic valve disease. However, surgical and transcatheter AVR carry risks of perioperative damage to the conduction system, requiring permanent pacemaker implantation. This can be explained by the anatomical proximity between the aortic valve annulus and the conduction system. Atrioventricular block and sinus node disease during or after AVR may be a consequence of periprocedural conduction system ischemia, direct surgical damage, local swelling, or mechanical pressure from the valve prosthesis. Risk factors associated with early pacemaker implantation include preoperative bundle branch block, older age, and a high burden of comorbidities.^1,2,3^ After surgical AVR, the prevalence of new permanent pacemaker implantation is 3% to 5%,^4,5,6^ whereas the prevalence after transcatheter AVR is 9% to 26%.^7,8,9,10,11^ However, prior studies investigating long-term clinical outcomes among patients who underwent permanent pacemaker implantation after AVR reported conflicting results.^4,12,13,14^ Furthermore, these studies are limited by their small patient numbers, short follow-up times, and single-center designs. Permanent pacemaker implantation is associated with pacemaker-induced heart failure, endocarditis, and lead-related complications.^15,16^ The association of permanent pacemaker implantation after AVR with long-term adverse outcomes is becoming increasingly important, especially when considering the use of transcatheter AVR in younger patients at lower risk of adverse surgical outcomes. Therefore, we performed a nationwide, population-based cohort study to investigate long-term prognosis after primary surgical AVR among patients who underwent postoperative permanent pacemaker implantation.

## Methods

We performed an observational, population-based cohort study. The study was approved by the Swedish Ethical Review Authority, and the requirement for informed consent was waived because data were deidentified. Reporting in this study conforms to the Strengthening the Reporting of Observational Studies in Epidemiology (STROBE) reporting guideline and Reporting of Studies Conducted Using Observational Routinely Collected Health Data (RECORD) guideline.^17^

### Setting

We included all patients who underwent surgical AVR in Sweden from 1997 to 2018. There are 8 centers that perform cardiac surgical procedures in Sweden. Follow-up ended on December 31, 2018.

### Data Sources

The Swedish Web-System for Enhancement and Development of Evidence-Based Care in Heart Disease Evaluated According to Recommended Therapies (SWEDEHEART) register^18,19^ was used to obtain the study cohort and baseline characteristics. Additional baseline characteristics, including socioeconomic data, were obtained from the Swedish National Patient Register^20^ and the Longitudinal Integrated Database for Health Insurance and Labor Market Studies register.^21^ Data on vital status, date of death, and cause of death were retrieved from the Swedish Cause of Death Register.^22^ Individual cross-linking of data between the national registries was possible owing to the unique personal identity number assigned to each Swedish citizen.^23^ The national registries used in this study were described in more detail previously.^24^

### Study Population and Exposure

We included all patients who underwent primary surgical AVR in Sweden from 1997 to 2018. Patients who died within 30 days of AVR, received a permanent pacemaker or implantable cardioverter defibrillator preoperatively, underwent concomitant surgical treatment for another valve or emergency surgical treatment (ie, within 24 hours from the decision to operate), had endocarditis preoperatively, or underwent surgical treatment owing to endocarditis were excluded. Patients with concomitant coronary artery bypass grafting or surgical treatment of ascending aorta were included. Exposure was defined as implantation of a permanent pacemaker or implantable cardioverter defibrillator within 30 days after AVR, as identified by *International Statistical Classification of Diseases and Related Health Problems, Tenth Revision *(*ICD-10*) codes (ie, FPE00, FPE10, FPE20, FPE26, FPF00, FPF10, FPF20, FPG10, FPG20, FPG30, and FPG33) from the National Patient Register.^20^ Patients were divided into 2 groups according to exposure: the pacemaker group and nonpacemaker group.

### Outcome

The primary outcome of this study was all-cause mortality. Secondary outcomes were heart failure hospitalization and endocarditis obtained from the National Patient Register,^20^ as well as cardiovascular death obtained from the Swedish Cause of Death Register.^22^

### Statistical Analysis

Baseline characteristics are shown as numbers and percentages for categorical variables and means and SDs for continuous variables. We generated propensity scores using generalized boosted regression modeling^25,26^ and calculated stabilized weights for inverse probability of treatment weighting to account for differences in baseline characteristics between the pacemaker and nonpacemaker groups. All variables in Table 1 were included in the estimation of propensity scores. Standardized mean differences (SMDs) were used to assess the balance between groups, and an SMD of less than 10% was considered an ideal balance.^27^ Each patient contributed a follow-up time, in days, from the date of surgical treatment until the date of death, date of 1 of the secondary outcomes, or end of follow-up (ie, December 31, 2018), whichever came first. All analyses were conducted in the weighted sample. To account for the competing risk of death, flexible parametric survival models were used to estimate cause-specific hazards and cumulative incidence of cardiovascular death, heart failure, and endocarditis.^28^ Data management and statistical analyses were performed using R statistical software version 4.0.3 (R Project for Statistical Computing) and Stata statistical software version 16.1 (StataCorp) and included use of the stpm2 and standsurv commands.^29^ *P* values were 2-sided and obtained from likelihood ratio tests; *P* < .05 was considered statistically significant. Data were analyzed from October through December 2020.

**Table 1.** Baseline Patient Characteristics

Characteristic	Unweighted patient population, No. (%)			Weighted patient population, No. (%)^a^		
	No pacemaker (n = 24 134)	Pacemaker (n = 849)	SMD	No pacemaker (n = 24 123.0)	Pacemaker (n = 714.4)	SMD
Age, mean (SD), y	69.7 (10.8)	69.4 (11.4)	0.026	69.7 (10.8)	69.8 (10.5)	0.006
Sex						
Women	8906 (36.9)	303 (35.7)	0.025	8901.8 (36.9)	258.6 (36.2)	0.014
Men	15 228 (63.1)	546 (64.3)		15 221.2 (63.1)	455.8 (63.4)	
Non-Nordic birth region	1473 (6.1)	42 (4.9)	0.051	1466.3 (6.1)	33.7 (4.7)	0.060
Educational level, y						
<10	10 458 (43.8)	347 (41.4)	0.090	10 434.6 (43.8)	308.7 (43.6)	0.032
10-12	8975 (37.6)	352 (42.0)		8996.4 (37.7)	275.5 (38.9)	
>12	4419 (18.5)	140 (16.7)		4410.6 (18.5)	123.4 (17.4)	
Disposable household income by quartile						
1 (low)	6026 (25.0)	219 (25.8)	0.083	6026.1 (25.0)	176.6 (24.7)	0.031
2	6054 (25.1)	191 (22.5)		6032.0 (25.0)	171.8 (24.0)	
3	6042 (25.0)	203 (23.9)		6033.7 (25.0)	178.9 (25.0)	
4 (high)	6008 (24.9)	236 (27.8)		6027.1 (25.0)	187.1 (26.2)	
Married	15 139 (62.7)	506 (59.6)	0.064	15 114.1 (62.7)	448.1 (62.7)	0.002
BMI						
<18.5	220 (1.0)	8 (1.0)	0.087	221.0 (1.0)	3.3 (0.5)	0.061
18.5-25	7392 (34.3)	293 (38.4)		7412.4 (34.4)	227.4 (35.2)	
>25	13965 (64.7)	462 (60.6)		13 940.2 (64.6)	415.6 (64.3)	
Atrial fibrillation	3907 (16.2)	157 (18.5)	0.061	3918.4 (16.2)	120.1 (16.8)	0.015
Heart failure	4879 (20.2)	221 (26.0)	0.138	4913.4 (20.4)	143.6 (20.1)	0.007
Left ventricular ejection fraction (%)						
>50	14 004 (74.7)	484 (67.4)	0.184	13 994.2 (74.4)	414.1 (73.9)	0.019
30-50	3906 (20.8)	176 (24.5)		3936.2 (20.9)	121.4 (21.7)	
<30	848 (4.5)	58 (8.1)		870.0 (4.6)	25.0 (4.5)	
Chronic obstructive pulmonary disease	2181 (9.0)	62 (7.3)	0.063	2171.0 (9.0)	54.1 (7.6)	0.052
Diabetes	4552 (18.9)	159 (18.7)	0.003	4550.2 (18.9)	129.4 (18.1)	0.019
eGFR, mL/min/1.73 m^2^						
>60	16 326 (73.4)	560 (71.3)	0.055	16 311.4 (73.4)	488.0 (74.0)	0.029
45-59	3974 (17.9)	157 (20.0)		3985.8 (17.9)	118.6 (18.0)	
30-44	1547 (7.0)	54 (6.9)		1546.8 (7.0)	43.1 (6.5)	
<30	382 (1.7)	14 (1.8)		382.0 (1.7)	9.4 (1.4)	
Preoperative dialysis	187 (0.9)	5 (0.7)	0.023	186.2 (0.8)	3.3 (0.5)	0.041
Prior myocardial infarction	3495 (14.5)	121 (14.3)	0.007	3495.9 (14.5)	95.2 (13.3)	0.034
Prior percutaneous coronary intervention	1963 (8.1)	63 (7.4)	0.027	1960.8 (8.1)	49.6 (6.9)	0.045
Peripheral vascular disease	2762 (11.4)	114 (13.4)	0.060	2770.1 (11.5)	83.4 (11.7)	0.006
Hypertension	10 664 (44.2)	400 (47.1)	0.059	10 679.9 (44.3)	328.4 (46.0)	0.034
Hyperlipidemia	4424 (18.3)	157 (18.5)	0.004	4423.0 (18.3)	133.7 (18.7)	0.010
Prior stroke	2380 (9.9)	89 (10.5)	0.021	2383.3 (9.9)	68.6 (9.6)	0.009
History of cancer	3162 (13.1)	130 (15.3)	0.063	3172.0 (13.1)	103.1 (14.4)	0.037
Alcohol dependence	548 (2.3)	15 (1.8)	0.036	546.0 (2.3)	11.0 (1.5)	0.053
Liver disease	294 (1.2)	5 (0.6)	0.067	290.8 (1.2)	4.8 (0.7)	0.056
Prior bleeding event	1684 (7.0)	54 (6.4)	0.025	1683.8 (7.0)	46.0 (6.4)	0.022
Bioprosthesis	17 843 (73.9)	630 (74.2)	0.006	17 840.7 (74.0)	525.0 (73.5)	0.011
Coronary artery bypass grafting	9103 (37.7)	271 (31.9)	0.122	9062.9 (37.6)	264.7 (37.1)	0.011
Isolated aortic valve replacement	13 155 (54.5)	496 (58.4)	0.079	13 176.7 (54.6)	386.8 (54.1)	0.010
Year of surgical treatment						
1997-2002	5513 (22.8)	138 (16.3)	0.211	5460.6 (22.6)	160.9 (22.5)	0.058
2003-2008	6186 (25.6)	200 (23.6)		6175.1 (25.6)	178.3 (25.0)	
2009-2013	6260 (25.9)	229 (27.0)		6260.7 (26.0)	173.7 (24.3)	
2014-2018	6175 (25.6)	282 (33.2)		6226.7 (25.8)	201.5 (28.2)	

Abbreviations: BMI, body mass index (calculated as weight in kilograms divided by height in meters squared); eGFR, estimated glomerular filtration rate; SMD, standardized mean difference.

^a^ Inverse probability of treatment weighting was used. The overall numbers of patients in each group are not necessarily integers owing to inverse probability of treatment weighting.

The following variables had missing data: left ventricular ejection fraction (5507 patients [22.0%]), estimated glomerular filtration rate (1969 patients [7.9%]), and educational level (292 patients [1.2%]). Missing data were handled by constructing weights to balance the groups in terms of missing data.^25,26^

## Results

Among 24 983 patients who underwent primary surgical AVR from 1997 to 2018 in Sweden, 849 patients (3.4%) underwent permanent pacemaker implantation within 30 days after surgical treatment and 24 134 patients (96.6%) did not receive pacemakers in that time. The mean (SD) age of the total study population was 69.7 (10.8) years, and there were 9209 patients were women (36.9%). Patients who underwent pacemaker implantation had an increased frequency of preoperative heart failure (221 patients [26.0%] vs 4879 patients [20.2%]; SMD, 0.138) and decreased frequency of concomitant coronary artery bypass grafting (271 patients [31.9%] vs 9103 patients [37.7%]; SMD, 0.122); however, in general, the groups were similar in terms of their baseline characteristics. After weighting, 24 123.0 patients without pacemakers and 714.4 patients with pacemakers (numbers of patients are not necessarily integers owing to inverse probability of treatment weighting) were balanced (eg, 8901.8 women without pacemakers [36.9%] vs 258.6 women with pacemakers [36.2%]; SMD, 0.014 and mean (SD) age, 69.7 (10.8) years among patients without pacemakers vs 69.8 (10.5) years among patients with pacemakers; SMD, 0.006) (Table 1; eFigure 1 in the Supplement). Baseline characteristics before and after weighting are shown in Table 1. The number of surgical procedures and the rate of permanent pacemaker implantation procedures per year are shown in eFigure 2 and eFigure 3, respectively, in the Supplement. Follow-up data were complete for all patients.

### Survival

**Figure 1.**
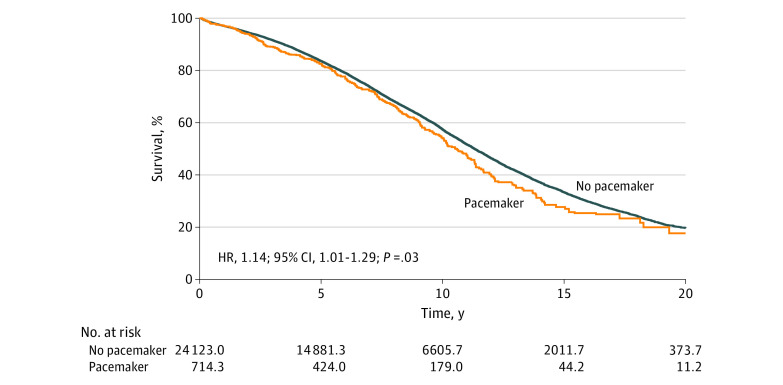
Survival After Aortic Valve Replacement Curves indicate Kaplan-Meier estimated survival after inverse probability of treatment weighting; HR, hazard ratio.

**Figure 2.**
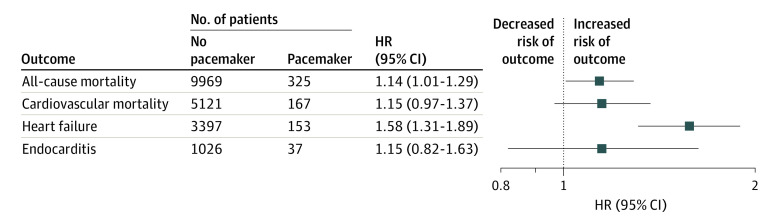
Risk Associated With Permanent Pacemakers After Aortic Valve Replacement HR indicates hazard ratio.

During a mean (SD) follow-up period of 7.3 (5.0) years (maximum follow-up, 22.0 years), 9969 patients (41.3%) died in the nonpacemaker group, while 325 patients (38.3%) died in the pacemaker group. The total follow-up time was 181 530 patient-years. At 10 years and 20 years after surgical treatment, the Kaplan-Meier estimated survival rates were 52.8% and 18.0% in the pacemaker group, respectively, and 57.5% and 19.6% in the nonpacemaker group, respectively. Kaplan-Meier estimated survival curves in the weighted cohort are illustrated in Figure 1. All-cause mortality rate was statistically significantly increased in the pacemaker group compared with the nonpacemaker group (hazard ratio [HR], 1.14; 95% CI, 1.01-1.29; *P* = .03) (Figure 2). The cumulative incidences for all-cause death 10 years and 20 years after AVR were 43.0% (95% CI, 42.3%-43.7%) and 80.9% (95% CI, 79.9%-81.2%) in the nonpacemaker group and 47.4% (95% CI, 43.5%-51.6%) and 84.9% (95% CI, 81.5%-88.5%) in the pacemaker group, respectively. The absolute risk differences 10 years and 20 years after AVR were 4.4% (95% CI, 0.3%-8.5%) and 4.0% (95% CI, 0.6%-7.5%), respectively (Table 2). We found no statistically significant differences between men and women in long-term outcomes associated with permanent pacemaker implantation. In the subset of patients who underwent isolated AVR, findings were similar to those in the total study population.

**Table 2.** Risks Associated With Permanent Pacemakers After AVR

	Incidence rate per 100 person-y (95% CI)	Cumulative incidence, % (95% CI)			
		5 y	10 y	15 y	20 y
All-cause death					
No pacemaker	5.7 (5.6 to 5.8)	16.5 (16.0 to 16.9)	43.0 (42.3 to 43.7)	66.2 (65.4 to 67.0)	80.9 (79.9 to 81.9)
Pacemaker	6.3 (5.7 to 7.0)	18.6 (16.7 to 20.7)	47.4 (43.5 to 51.6)	71.0 (66.9 to 75.4)	84.9 (81.5 to 88.5)
Absolute risk difference	NA	2.1 (0.1 to 4.1)	4.4 (0.3 to 8.5)	4.9 (0.5 to 9.2)	4.0 (0.6 to 7.5)
Cardiovascular death					
No pacemaker	2.9 (2.8 to 3.0)	8.3 (8.0 to 8.7)	21.7 (21.1 to 22.3)	34.1 (33.3 to 34.9)	42.0 (41.0 to 43.1)
Pacemaker	3.3 (2.8 to 3.8)	9.5 (8.1 to 11.1)	24.1 (20.9 to 27.9)	36.9 (32.4 to 42.1)	44.4 (39.3 to 50.2)
Absolute risk difference	NA	1.2 ( −0.4 to 2.7)	2.4 ( −1.1 to 6.0)	2.8 ( −2.1 to 7.7)	2.4 ( −3.1 to 7.9)
Heart failure					
No pacemaker	2.0 (2.0 to 2.1)	7.0 (6.7 to 7.3)	15.2 (14.7 to 15.7)	21.7 (21.0 to 22.4)	25.9 (25.0 to 26.8)
Pacemaker	3.1 (2.7 to 3.8)	10.8 (9.1 to 12.8)	22.6 (19.3 to 26.5)	31.2 (26.9 to 36.2)	36.3 (31.4 to 41.8)
Absolute risk difference	NA	3.8 (1.9 to 5.6)	7.4 (3.8 to 11.1)	9.6 (4.9 to 14.2)	10.4 (5.2 to 15.6)
Endocarditis					
No pacemaker	0.59 (0.56 to 0.63)	3.0 (2.8 to 3.2)	4.7 (4.4 to 5.0)	5.8 (5.4 to 6.1)	6.4 (6.0 to 6.8)
Pacemaker	0.70 (0.50 to 1.00)	3.4 (2.5 to 4.8)	5.3 (3.8 to 7.4)	6.4 (4.6 to 8.9)	7.0 (5.0 to 9.7)
Absolute risk difference	NA	0.4 ( −0.7 to 1.6)	0.6 ( −1.2 to 2.4)	0.6 ( −1.5 to 2.8)	0.6 ( −1.7 to 2.9)

Abbreviations: AVR, aortic valve replacement; NA, not applicable.

### Cardiovascular Death

**Figure 3.**
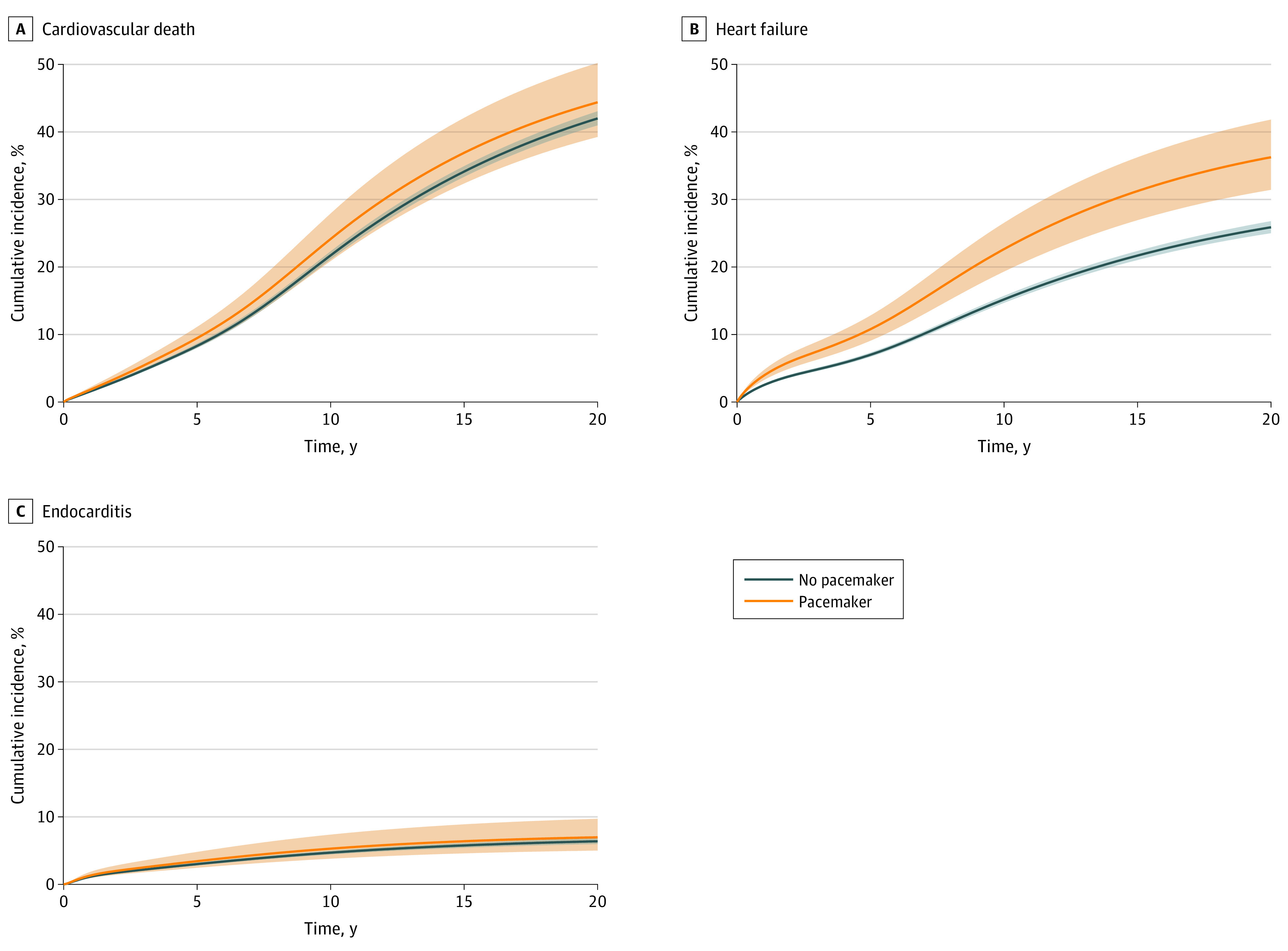
Incidence of Cardiovascular Death, Heart Failure, and Endocarditis After Aortic Valve Replacement

Among 325 patients who died during the study period in the pacemaker group, 167 patients (51.4%) died from cardiovascular causes, and among 9969 patients who died in the nonpacemaker group, 5121 patients (51.4%) died from cardiovascular causes. At 10 years and 20 years after surgical treatment, the cumulative incidences of cardiovascular death were 24.1% (95% CI, 20.9% to 27.9%) and 44.4% (95% CI, 39.3% to 50.2%) in the pacemaker group, respectively, and 21.7% (95% CI, 21.1% to 22.3%) and 42.0% (95% CI, 41.0% to 43.1%) in the nonpacemaker group, respectively. The cumulative incidence of cardiovascular death is illustrated in Figure 3. No association was found between permanent pacemaker implantation and cardiovascular death (HR, 1.15; 95% CI, 0.97 to 1.37; *P* = .10) (Figure 2). The absolute risk differences at 10 years and 20 years after AVR were 2.4% (95% CI, −1.1% to 6.0%) and 2.4% (95% CI, −3.1% to 7.9%), respectively. The cumulative incidence and absolute risk differences at 5 years, 10 years, 15 years, and 20 years for cardiovascular death are shown in Table 2.

### Heart Failure Hospitalization

During follow-up, 153 patients (18.0%) in the pacemaker group and 3397 patients (14.1%) in the nonpacemaker group were hospitalized for heart failure. At 5 years, 10 years, and 15 years after surgical treatment, the cumulative incidences of hospitalization for heart failure were 10.8% (95% CI, 9.1%-12.8%), 22.6% (95% CI, 19.3%-26.5%), and 31.2% (95% CI, 26.9%-36.2%) in the pacemaker group, respectively, and 7.0% (95% CI, 6.7%-7.3%), 15.2% (95% CI, 14.7%-15.7%), and 21.7% (95% CI, 21.0%-22.4%) in the nonpacemaker group, respectively. The cumulative incidence of heart failure hospitalization is illustrated in Figure 3. The risk of heart failure hospitalization was statistically significantly increased in the pacemaker group compared with the nonpacemaker group (HR, 1.58; 95% CI, 1.31-1.89; *P* < .001) (Figure 2). The absolute risk differences at 5 years, 10 years, and 15 years after AVR were 3.8% (95% CI, 1.9%-5.6%), 7.4% (95% CI, 3.8%-11.1%), and 9.6% (95% CI, 4.9%-14.2%), respectively. The cumulative incidences and absolute risk differences at 5 years, 10 years, 15 years, and 20 years for heart failure hospitalization are shown in Table 2.

### Endocarditis

During follow-up, 37 patients (4.4%) in the pacemaker group and 1026 patients (4.3%) in the nonpacemaker group had endocarditis. At 10 years and 20 years after surgical treatment, the cumulative incidences of endocarditis were 5.3% (95% CI, 3.8% to 7.4%) and 7.0% (95% CI, 5.0% to 9.7%) in the pacemaker group, respectively, and 4.7% (95% CI, 4.4% to 5.0%) and 6.4% (95%, CI 6.0% to 6.8%) in the nonpacemaker group, respectively. The cumulative incidence of endocarditis is illustrated in Figure 3. No association was found between permanent pacemaker implantation and endocarditis (HR, 1.15; 95% CI, 0.82 to 1.63; *P* = .42) (Figure 2). The absolute risk differences at 10 years and 20 years after AVR were 0.6% (95% CI, −1.2% to 2.4%) and 0.6% (95% CI, −1.7% to 2.9%), respectively. The cumulative incidences and absolute risk differences at 5 years, 10 years, 15 years, and 20 years for endocarditis are shown in Table 2.

## Discussion

In this nationwide, population-based cohort study, we observed an increased risk of death and heart failure hospitalization among patients who underwent permanent pacemaker implantation after AVR compared with those who did not undergo this implantation after AVR. We found no association between permanent pacemaker implantation and risk of endocarditis.

Particular strengths of our study include the nationwide and population-based design, as well as the large number of patients and long-term follow up. Because of the accuracy of Swedish national registries, our study provided high-quality data, and follow-up data were complete for all patients. Greason et al^4^ analyzed 5842 patients who underwent surgical AVR at the Mayo Clinic (Rochester, Minnesota) from 1993 to 2014. Permanent pacemakers were implanted in 2.5% of patients within 30 days after surgical treatment. The mortality rate at 10 years was 65% in the pacemaker group, and worse long-term survival was noted among patients who underwent permanent pacemaker implantation compared with those who did not (HR, 1.49; 95% CI, 1.20-1.84; *P* < .001). In our study, we also observed worse long-term survival among patients who underwent permanent pacemaker implantation after AVR (HR, 1.14; 95% CI, 1.01-1.29; *P* = .03), and 10-year mortality was 47.4% among patients who underwent permanent pacemaker implantation. The decreased 10-year mortality in our study may be associated with a younger patient cohort with a lower degree of concomitant coronary artery bypass grafting. Furthermore, Greason et al did not exclude patients who underwent nonelective surgical treatment or had undergone prior cardiac surgical treatment. Nevertheless, our results support the findings presented by Greason et al and provide robust data obtained from a nationwide and contemporary patient cohort consisting of almost 25 000 patients. Furthermore, our study adds information about heart failure hospitalization, endocarditis, and cardiovascular death among patients who underwent permanent pacemaker implantation after surgical AVR.

In contrast to our results, other studies found no association between postoperative pacemaker implantation and long-term mortality.^12,14^ Bagur et al^12^ found no statistically significant difference in survival between patients who received pacemakers and those who did not among 780 patients who underwent isolated surgical AVR at a single center in Canada. However, the authors reported over a follow-up period of only 5 years. Our findings suggest that the association of permanent pacemaker implantation with adverse outcomes becomes clinically apparent over a longer follow-up period. Furthermore, patients in Bagur et al were almost 10 years older than those in our study. It can be hypothesized that older patients are more likely to die from other causes before complications related to their pacemakers become associated with negative clinical outcomes.

Conventional right ventricular pacing is associated with adverse left ventricular remodeling and decreased left ventricular ejection fraction compared with biventricular pacing.^16^ This finding may be associated with the dyssynchronous systolic left ventricular function observed with right ventricular pacing,^30^ which may lead to reduced left ventricular function and heart failure.^16^ This theory corresponds well with our results because we observed an increased risk of heart failure hospitalization among patients who underwent new permanent pacemaker implantation (HR, 1.58; 95% CI, 1.31-1.89; absolute risk difference, 9.6% at 15 years), suggesting a clinically and statistically significant association. However, because we did not have information about the right ventricular pacing burden at follow-up, this hypothesis could not be confirmed. We found an increased risk of cardiovascular death among patients who underwent permanent pacemaker implantation after AVR, but this was not statistically significant. This may have been associated with the low number of events, and we believe that a larger study may indeed find an increased risk of cardiovascular death among patients who undergo permanent pacemaker implantation after AVR.

Permanent pacemaker implantation can have several complications, including lead-related complications; traumatic complications, such as pneumothorax and pericardial effusion; pocket complications; and infection. In a multicenter study^15^ from the Netherlands, the complication rate after permanent pacemaker implantation at 5 years was approximately 20%, and permanent pacemaker implantation after AVR is associated with longer hospital stays and increased costs.^6,12^ Our study findings suggest that permanent pacemaker implantation after AVR is also associated with an increased risk of all-cause mortality and heart failure hospitalization in the long term. However, it is reassuring to note that we found no clinically relevant association between permanent pacemaker implantation and risk of endocarditis. All available evidence suggests an increased risk of adverse clinical outcomes among patients who underwent permanent pacemaker implantation after AVR and underscores the necessity to decrease rates of pacemaker implantation or avoid the procedure after AVR.

In some studies, 40% to 45% of patients who underwent permanent pacemaker implantation after AVR were dependent on pacing after a few years of follow-up.^14,31^ This raises questions about the optimal timing for permanent pacemaker implantation after AVR and whether it should be postponed beyond the 7 days currently recommended after cardiac surgical treatment to determine if rhythm disturbances are temporary or permanent.^32^ Furthermore, the implantation of some surgical aortic valve prostheses is associated with an increased risk of postoperative pacemaker dependency.^33^ The risks of permanent pacemaker implantation should be considered when choosing the optimal valve prosthesis for each patient.

The prevalence of permanent pacemaker implantation after transcatheter AVR is consistently increased compared with the prevalence after surgical AVR.^10,34^ Although our results cannot be directly generalized to patients who underwent transcatheter AVR, it is likely that our findings may be valid in transcatheter AVR patient populations. The results of our study are clinically relevant, especially in an era when transcatheter AVR is used among younger patients with lower surgical risk. Younger patients have a longer life expectancy; therefore, the association of permanent pacemaker implantation with adverse outcomes becomes more relevant in this patient population. Thus, the risks associated with permanent pacemaker implantation after transcatheter AVR are becoming increasingly important.

### Limitations

This study has several limitations. Although we adjusted for a range of comorbidities and socioeconomic factors, there were factors that were unknown or unmeasured; thus, we did not adjust for them (ie, there was residual confounding). Examples of such factors are preoperative electrocardiographic characteristics and indications for pacemaker implantation. Thus, this study allows for demonstrating associations rather than causality. Furthermore, we could not discriminate between patients with a high pacing burden and low or no pacing dependency during follow-up. While we examined several clinically important outcomes, our study did not investigate other central aspects of well-being, such as quality of life or functional capacity.

## Conclusions

We observed an increased risk of all-cause mortality and heart failure hospitalization among patients who underwent permanent pacemaker implantation after surgical AVR. We found no association between permanent pacemaker implantation and risk of endocarditis. Our findings are important to consider, especially in an era when transcatheter AVR is used among younger patients at lower risk of adverse surgical outcomes. These findings suggest that future research should investigate how to avoid permanent pacemaker dependency after surgical and transcatheter AVR.

## 

## Notes

Supplement. **eFigure 1.** Absolute Standardized Differences Before (Hollow Circles) and After (Filled Circles) Inverse Probability of Treatment Weighting in Total Study Population **eFigure 2.** No. Operations per Year in Sweden From 1997 to 2018 **eFigure 3.** Rate of Permanent Pacemaker Implantation in Sweden From 1997 to 2018
