# Supplementary material for: Long-term Outcomes Associated With Permanent Pacemaker Implantation After Surgical Aortic Valve Replacement
Source: JAMA Netw Open. 2021 Jul 13;4(7):e2116564. doi: 10.1001/jamanetworkopen.2021.16564 (PMC8278270; doi:10.1001/jamanetworkopen.2021.16564)
Supplement: Supplement. — eFigure 1. Absolute Standardized Differences Before (Hollow Circles) and After (Filled Circles) Inverse Probability of Treatment Weighting in Total Study Population eFigure 2. No. Operations per Year in Sweden From 1997 to 2018 eFigure 3. Rate of Permanent Pacemaker Implantation in Sweden From 1997 to 2018 [file jamanetwopen-e2116564-s001.pdf]

Supplemental Online Content

Glaser N, Persson M, Dalén M, Sartipy U. Long-term outcomes associated with permanent pacemaker implantation after surgical aortic valve replacement. *JAMA Netw Open*. 2021;4(7):e2116564. doi:10.1001/jamanetworkopen.2021.16564

eFigure 1. Absolute Standardized Differences Before (Hollow Circles) and After (Filled Circles) Inverse Probability of Treatment Weighting in Total Study Population
eFigure 2. No. Operations per Year in Sweden From 1997 to 2018
eFigure 3. Rate of Permanent Pacemaker Implantation in Sweden From 1997 to 2018

This supplemental material has been provided by the authors to give readers additional information about their work.

© 2021 Glaser N et al. *JAMA Network Open.*

eFigure 1. Absolute Standardized Differences Before (Hollow Circles) and After (Filled Circles) Inverse Probability of Treatment Weighting in Total Study Population

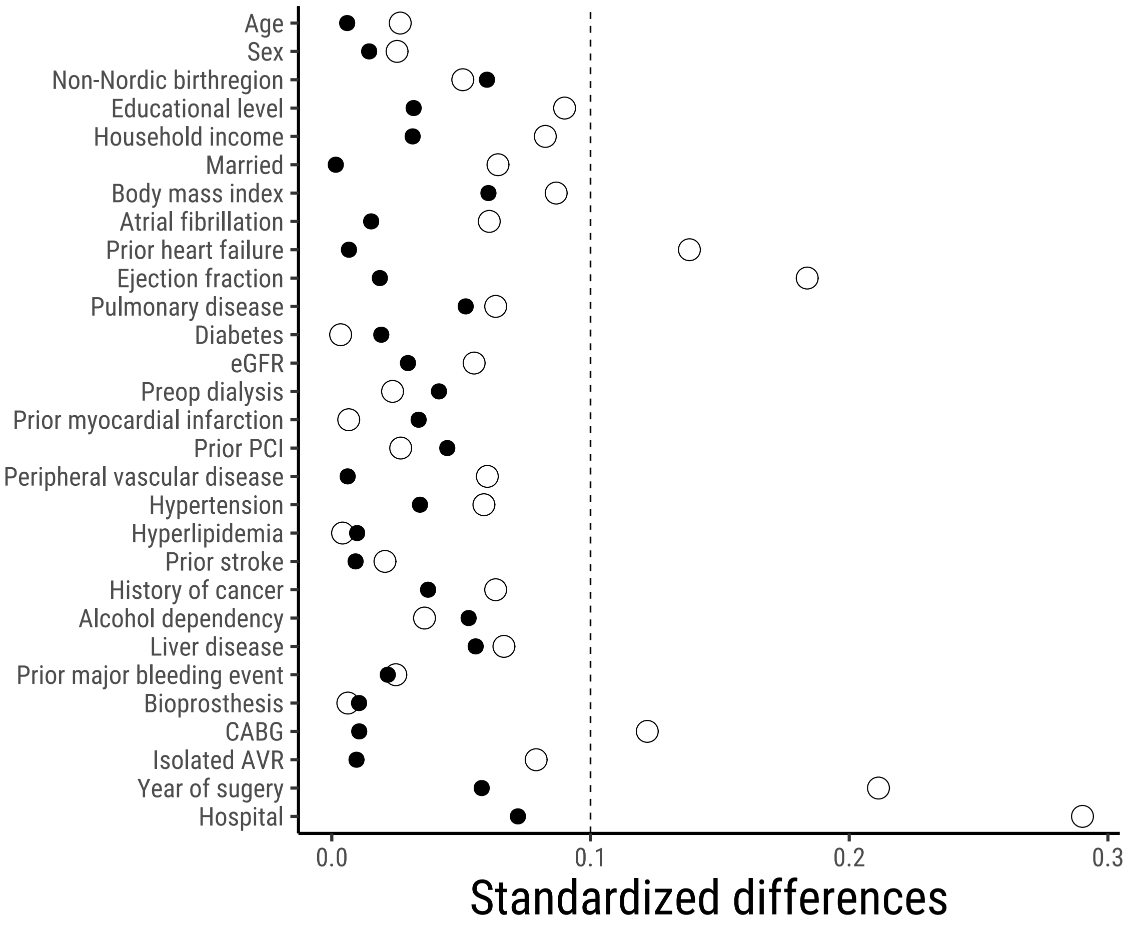

© 2021 Glaser N et al. *JAMA Network Open.*

eFigure 2. No. Operations per Year in Sweden From 1997 to 2018

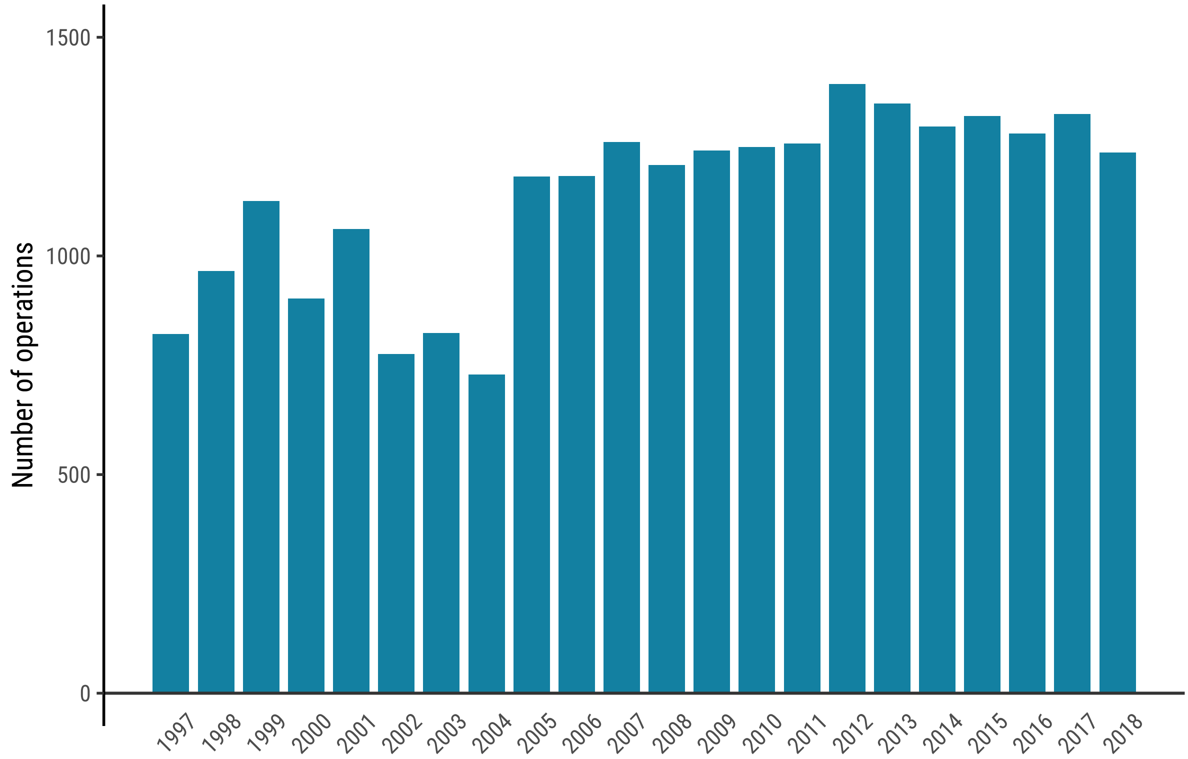

© 2021 Glaser N et al. *JAMA Network Open.*

eFigure 3. Rate of Permanent Pacemaker Implantation in Sweden From 1997 to 2018

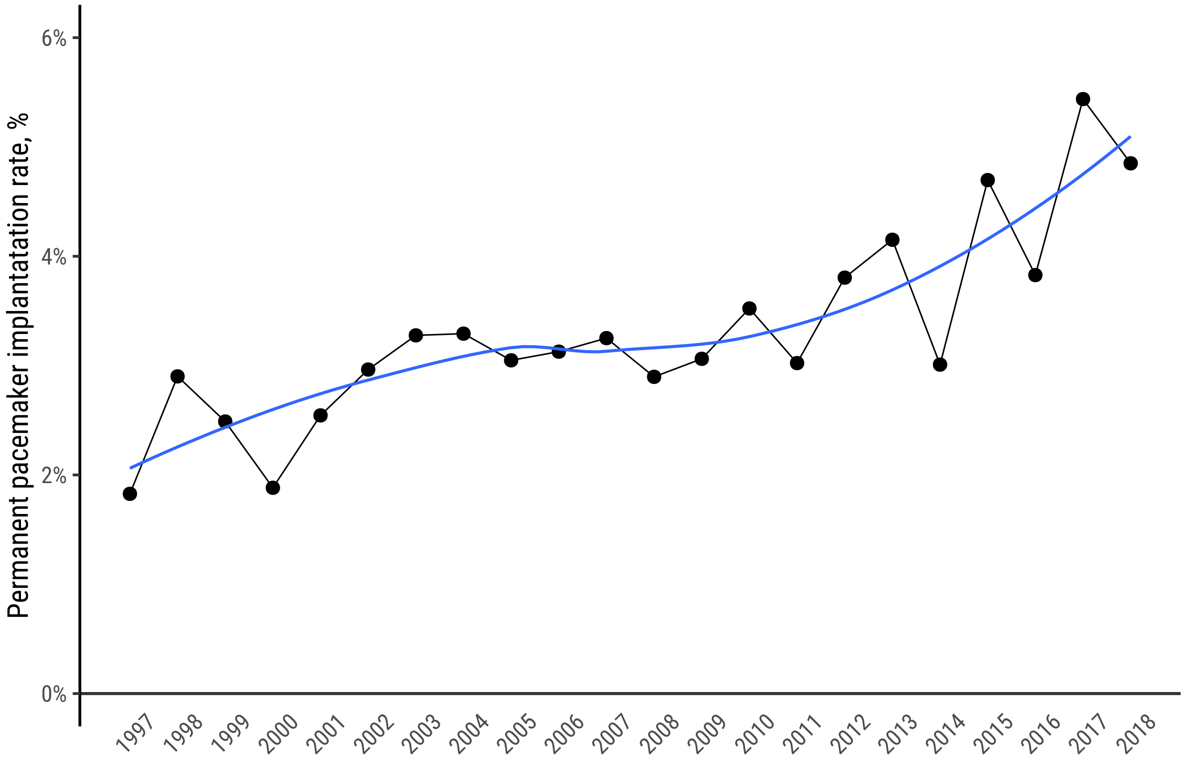

© 2021 Glaser N et al. *JAMA Network Open.*
